# Large-Scale Isolation of Microsatellites from Chinese Mitten Crab *Eriocheir sinensis* via a Solexa Genomic Survey

**DOI:** 10.3390/ijms131216333

**Published:** 2012-12-03

**Authors:** Liang-Wei Xiong, Qun Wang, Gao-Feng Qiu

**Affiliations:** 1 Key laboratory of Freshwater Aquatic Genetic Resources Certificated by Ministry of Agriculture, College of Life Science, Shanghai Ocean University, 999 Hucheng Huan Road, Shanghai 201306, China; E-Mail: xlwei2002@163.com; 2 Jiangsu Animal Husbandry & Veterinary College, Jiangsu 225300, China; 3 College of Life Science, East China Normal University, Shanghai 200062, China; E-Mail: qwang@bio.ecnu.edu.cn

**Keywords:** microsatellite marker, *Eriocheir sinensis*, solexa sequencing

## Abstract

Microsatellites are simple sequence repeats with a high degree of polymorphism in the genome; they are used as DNA markers in many molecular genetic studies. Using traditional methods such as the magnetic beads enrichment method, only a few microsatellite markers have been isolated from the Chinese mitten crab *Eriocheir sinensis*, as the crab genome sequence information is unavailable. Here, we have identified a large number of microsatellites from the Chinese mitten crab by taking advantage of Solexa genomic surveying. A total of 141,737 SSR (simple sequence repeats) motifs were identified via analysis of 883 Mb of the crab genomic DNA information, including mono-, di-, tri-, tetra-, penta- and hexa-nucleotide repeat motifs. The number of di-nucleotide repeat motifs was 82,979, making this the most abundant type of repeat motif (58.54%); the second most abundant were the tri-nucleotide repeats (42,657, 30.11%). Among di-nucleotide repeats, the most frequent repeats were AC motifs, accounting for 67.55% of the total number. AGG motifs were the most frequent (59.32%) of the tri-nucleotide motifs. A total of 15,125 microsatellite loci had a flanking sequence suitable for setting the primer of a polymerase chain reaction (PCR). To verify the identified SSRs, a subset of 100 primer pairs was randomly selected for PCR. Eighty two primer sets (82%) produced strong PCR products matching expected sizes, and 78% were polymorphic. In an analysis of 30 wild individuals from the Yangtze River with 20 primer sets, the number of alleles per locus ranged from 2–14 and the mean allelic richness was 7.4. No linkage disequilibrium was found between any pair of loci, indicating that the markers were independent. The Hardy-Weinberg equilibrium test showed significant deviation in four of the 20 microsatellite loci after sequential Bonferroni corrections. This method is cost- and time-effective in comparison to traditional approaches for the isolation of microsatellites.

## 1. Introduction

Microsatellites or simple sequence repeats (SSRs), which are tandemly repeated units of one to six nucleotides, have been abundant in all prokaryotic and eukaryotic genomes analysed to date [1,2]. They are evenly distributed throughout genomes and are usually characterized by a high degree of length polymorphism, which makes them one of the most popular genetic markers for a wide range of applications including genetic mapping, marker-assisted selection breeding (MAS), genetic diversity studies, population structure analysis, gene flow and germplasm conservation studies [3–6]. However, a major drawback of the application of microsatellite markers is that they need to be isolated *de novo* from most organisms being examined for the first time.

Traditionally, the isolation of SSR markers has relied on the screening of genomic libraries using repetitive probes and sequencing of positive clones to develop SSR primers [7]. However, most of these steps are difficult, time-consuming, and relatively inefficient. Next-generation sequencing (NGS) technologies that speed up the process to generate a large number of sequences have been used recently to isolate SSR markers in studies of non-model animals [8,9], plants [10–12] and fishes [13–15].

The Chinese mitten crab, *Eriocheir sinensis*, is a euryhaline brachyuran with a native range extending from the eastern Pacific coast of China to the Korean Peninsula [16]. In China, the basic production technology of mitten crab populations has had a long history, and today, it is one of the most economically important indigenous organisms in freshwater aquaculture with an annual aquaculture production of 570,000 mt in 2009, valued at 4.0 billion USD, according to the State of World Fisheries and Aquaculture from FAO [17]. Unfortunately, like many other cultured decapod species the mitten crab has not been completely domesticated and many broodstock crabs are collected from wild populations. To develop a strain with good performance, conventional selective breeding programs of the crab have been conducted for several years. Because the selection was performed based mainly on phenotypic assessment and because the breeding cycle is long, the breeding programs of the mitten crab have been inefficient. As verified in many cultured species, molecular markers introducing selection (*i.e.*, MAS) are required to accelerate the course of the crab breeding [18]. Usually, MAS depends on a high-resolution genetic linkage map for various purposes including characterization of quantitative trait loci (QTL) [19]. Development of a large number of sequence-based genetic markers, such as microsatellites, is an essential step for MAS and linkage map construction. However, karyotypic analysis has shown that the diploid chromosome number in the mitten crab is large (2*n* = 146) [20]. It is estimated that hundreds of SSRs are required for construction of a high-density linkage map. To date, approximately 47 microsatellite markers have been developed [21–24], which is inadequate for construction of a linkage map. In this study, we used the Solexa sequencing technology for the whole genomic DNA survey of the Chinese mitten crab, in order to isolate polymorphic microsatellites on a large-scale for its linkage map construction.

## 2. Results

### 2.1. Genome Survey and Assembly

Solexa genomic surveying produced a total of 76.27 Gb of raw genomic data. We assembled the short reads using SOAP *de novo*, a genome assembler developed specifically for use with next-generation short-read sequences [25]. After excluding the data from poor libraries (reads with more than 10% of Q < 20 bases) and filtering low-quality sequences (reads with ambiguous bases “N”), 56.20 Gb reads remained as high-quality reads for *de novo* assembly. Finally, 883 Mb of sequence data were obtained from 1,096,936 scaffolds with a length range from 0.1 kb to 10 kb.

### 2.2. Microsatellite Loci Discovery and Primer Pair Design

**Figure 1:**
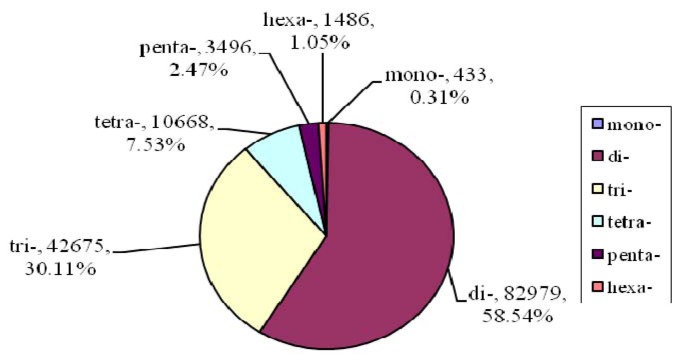
The percentages of mono-, di-, tri-, tetra-, penta- and hexa-nucleotide repeats in motif sequences from the Chinese mitten crab.

**Figure 2:**
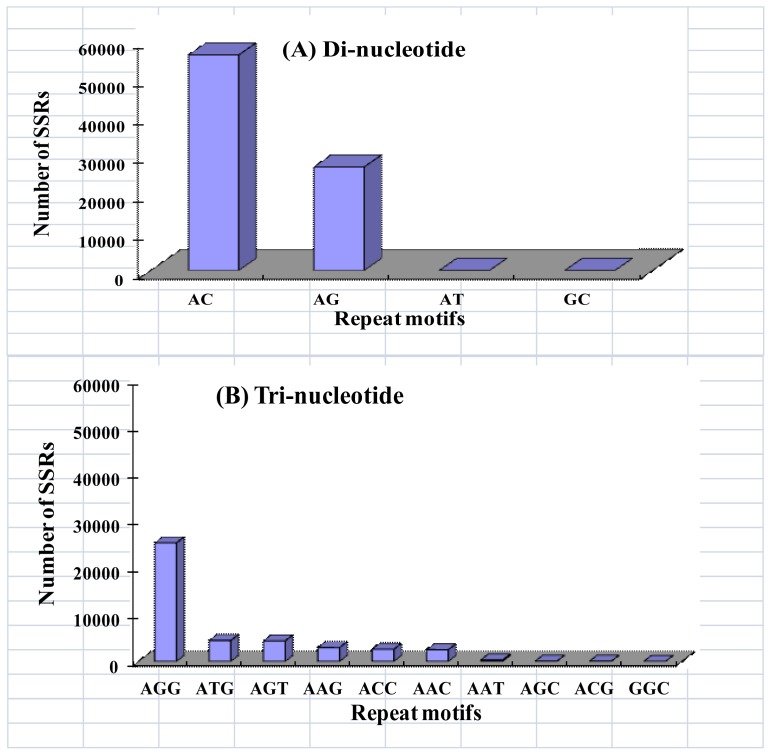
Observed counts of identified microsatellite loci for different repeat sequence motifs of (**A**) di- nucleotide and (**B**) tri-nucleotide repeats from the Chinese mitten crab.

**Figure 3:**
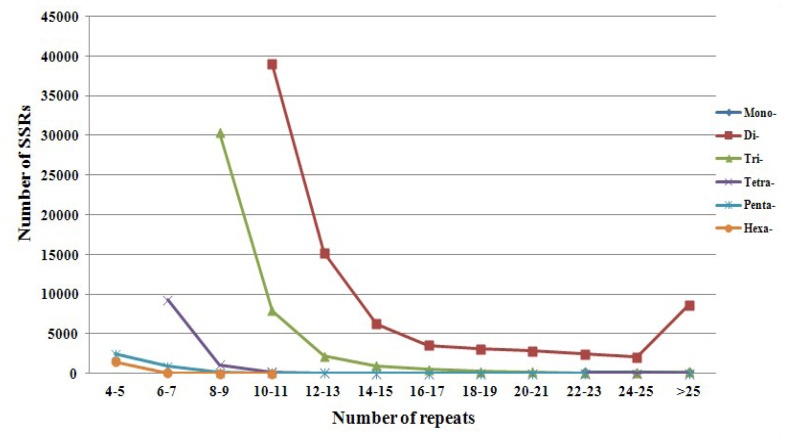
Observed number of microsatellites with mono-, di-, tri-, tetra-, penta- and hexa-nucleotide motifs in 883 Mb sequence.

The resultant 883 Mb of DNA sequence was analyzed to evaluate different types of perfect mono-, di-, tri-, tetra-, penta- and hexa-nucleotides. A total of 141,737 distinct microsatellite loci were identified. The SSR distribution density was approximately 161 loci per Mb. The most abundant type of repeat motif was a di-nucleotide (58.54%), followed by tri-nucleotide (30.11%), tetra-nucleotide (7.53%), penta-nucleotide (2.47%), hexa-nucleotide (1.05%), and mono-nucleotide (0.31%) repeat units (Figure 1). There were large differences in the relative abundance of special repeat motifs. As shown in Figure 2A, among the di-nucleotide sequences, the motif AC had the highest frequency, representing 67.55% of the sampled sequences, followed by AG (32.44%). Motifs AT and GC (<0.01% each) were comparatively rare. The most frequent tri-nucleotide was AGG (59.32%), whereas AAT (0.74%), AGC (0.11%), ACG (0.09%), and GCC (0.01%) were comparatively scarce (Figure 2B). The frequency distributions from mono- to hexa-nucleotide repeats were calculated and are shown in Figure 3. The bulk of repeat sequences were centralized in the domain composed of low copy number, and fewer sequences were seen with increasing copy number. Among the di-nucleotide repeat sequences, repeats with 10–11 copies were the most common (33.28%); among tri-nucleotide repeat sequences, repeats with 8–9 copies were the most common (22.28%). The size of each repeat sequence was determined by the copy number of its repeat unit (Table 1).

To estimate the number of loci that represented promising candidates for PCR amplification-based scoring of microsatellite length variation, we screened the loci to determine which of them contained suitable flanking PCR primer sites; we referred to such loci as “potentially amplifiable loci” or PAL. We identified 15,125 PAL and designed their corresponding primer pairs, which represented 10.67% of microsatellite loci identified.

### 2.3. SSR Validation and Population Genetic Analysis

A subset of 100 PAL was selected for validation. Primers were designed for these loci and tested using the genomic DNA of a panel of five individuals. Eighty-two primer sets (82%) successfully yielded amplicons matching the expected sizes although they contained some nonspecific bands, and eighteen primer pairs did not give any amplification product. Of the eighty-two primer sets, four sets generated monomorphic products in all the tested individuals. A panel of 20 SSRs was used for further polymorphism testing in 30 individuals from a wild population. The primer sequences, repeat motifs, annealing temperatures, number of alleles, PCR ranges and the heterozygosity for the 20 new microsatellite loci are summarized in Table 2. The amplification results showed that all the loci were polymorphic. The number of alleles per locus varied from 2 to 14 with an average of 7.4. No linkage disequilibrium was found between any pair of loci (*p* > 0.05 indicating that the markers were independent. The Hardy-Weinberg equilibrium (HWE) test, indicating the deviation from the expected heterozygosity, showed significant deviation in four (*Eri*3, *Eri*8, *Eri*11 and *Eri*14) of the 20 loci in the wild population after sequential Bonferroni corrections. Null alleles were presumed in five (*Eri*3, *Eri*6, *Eri*8, *Eri*14 and *Eri*16) of the 20 loci. Expected heterozygosities (*He*) ranged from 0.510 to 0.971 (mean: 0.800 ± 0.147) and observed heterozygosities (*Ho*) from 0.326 to 0.958 (mean: 0.689 ± 0.170).

## 3. Discussion

Prior to our study, less than 50 SSR markers had been developed in the mitten crab using conventional methods [21–23,26]. By taking advantage of the Solexa genomic survey here, we discovered an extensive set of 141,737 microsatellite loci in which 15,125 loci are PAL. Compared to the weeks or even months that would have been spent obtaining only tens of microsatellite loci by conventional methods, this process only took one month. This method consisting of a genomic survey is composed of only four steps: (i) isolation of genomic DNA; (ii) DNA sequencing and assembly; (iii) microsatellite loci discovery and primer design, and (iv) microsatellite verification [8]. No cloning or library screening is required. This new method targets all microsatellite repeat types (e.g., mono-, di-, tri-, tetra-, penta-, and hexa-nucleotide), while conventional methods require an SSR-enriched genomic library for screening a limited number of specific microsatellite motifs, and the choice of motif can have an effect on the variability detected [27]. Thus, the Solexa genomic survey is an effective method for large-scale isolation of microsatellite markers. Furthermore, millions of base pairs of genomic sequence are available in the survey, potentially providing a framework for further genomic analyses and a useful resource for gene research.

It is estimated that the size of crustacean genomes ranges from 55 Mb to 1800 Mb [28]. The cumulative length of the assembled sequences of the Chinese mitten crab genome reached 883 Mb and will provide important information about the mitten crab genomic organization of repeat sequences. The di-nucleotide repeats contributed to a major proportion of genome SSRs, while only a very small proportion was contributed by mono-, tri-, tetra-, penta- and hexa-nucleotide repeats (Figure 1). The number of di-nucleotide repeats was also the most prevalent in the Chinese shrimp [29], fruit fly [30], pufferfish [31], human [32], and plant [33]. Moreover, the di-nucleotide repeat class was the only class of repeats found in the genome of the prokaryote methanogenic archaeon (*Methanococcus jannaschii*) [34]. These data indicate that di-nucleotide repeats may have an important biological significance in the genesis and development of repeat sequences. Among the di-nucleotide repeats, AC was the most frequent motif in the crab genome as observed in many other eukaryotes [35], with the exception of plants in which AT was the most abundant motif [36]. Interestingly, the frequency of GC and AT di-nucleotide repeats was in less than 0.01% each of the crab genome (Figure 2A). This result is similar to that of most organisms including plants whose genome contains the rare GC repeat class [37]. One explanation of this GC suppression may be that the GC repeats present structural problems, and this point was exemplified with the association of a similar CCG repeat with the fragile X site on the human X chromosome [29]. Among the tri-nucleotide repeats, AGG (59.32%) was the most frequent in our dataset, as it was in the Japanese pufferfish [31]. AAT, AGC, AAG were the most frequent motifs in human, fruit fly and Chinese shrimp, respectively [29,30,32]. We hypothesized that the relative frequency of different types of tri-nucleotide motifs varied according to species. 1789 5,789

When a subset of 100 primer pairs from 15,125 PAL was tested, 78 of them were successfully amplified with polymorphisms from five individuals. When 20 microsatellite markers were selected for further polymorphism testing, all of them showed high diversity and variation among the 30 individuals tested. Adjusting for null alleles, the mean number of alleles per locus, *H*_E_ and *H*_O_ are 7.4, 0.800 and 0.689 respectively, demonstrating a relatively high genetic diversity within crab individuals. This is similar to reports from studies in other locations [21,23,24,38,39]; however, four microsatellite loci exhibited drastic departures from *HWE* as shown by the fact that *H*_E_ was apparently higher than *H*_O_ (Table 2). In this study, heterozygote deficiency at the four loci seems to be strong evidence for the deviations of *HWE*. Although there are several possible explanations for a deficiency of heterozygotes, here the consistent pattern across loci suggests that the individuals examined most likely do not originate from a single panmictic population (individuals in this study were sampled from the Yangtze River over quite a large geographical distance).

## 4. Experimental Section

### 4.1. Sample

A single specimen of Chinese mitten crab from one aquaculture farm in China was used as the sole source of tissue/DNA for a genomic DNA survey. The total genomic DNA was extracted from the muscle tissue using the standard proteinase K, phenol-chloroform procedure [40]. The genomic DNA concentration was quantified using an ultraviolet (UV) spectrophotometer (Nanodrop, Madison, WI, USA), and the DNA quality was assessed on a 0.8% agarose gel. In the population genetic analysis, the total DNA was extracted by the same method from ethanol-fixed tissues of 30 wild individuals who had been stored at the Key laboratory of Freshwater Aquatic Genetic Resources Certified by the Ministry of Agriculture, China.

### 4.2. DNA Sequencing

Approximately 1 mg of genomic DNA (>23 kb, OD260/OD280 ≈ 1.80) was subjected to Solexa sequencing analysis at Beijing Genomics Institute (BGI; Shenzhen, China) using whole-genome shotgun sequencing strategy and Illumina Genome Analyzer sequencing technology. Libraries with an insert size of 170 bp and 500 bp were prepared following the manufacturer’s instructions (Illumina, San Diego, CA, USA). After library preparation and quality control of the DNA samples, four lane (two 170 bp, two 500 bp) template DNA fragments were hybridized to the surface of flow cells on an Illumina Genome Analyzer II sequencer (GA2), amplified to form clusters, and sequenced following the standard Illumina protocol.

### 4.3. *De Novo* Assembly

The read sequence was aligned using the SOAP *de novo* software [25] with the default setting, which adopts the De Bruijin graph data structure to construct contigs [41]. The reads were then realigned to the contig sequence, and the paired-end relationship between the reads was transferred to linkage between contigs. Scaffolds starting with short paired-ends were constructed and then the scaffold process was iterated step-by-step using longer insert-size paired-ends. To fill the intra-scaffold gaps, the paired-end information was used to retrieve read pairs that had one read well-aligned on the contigs and another read located in the gap region. We then did a local assembly for the collected reads.

### 4.4. Identification of Microsatellite Loci

The assembled sequences were scanned for perfect mono-, di-, tri-, tetra-, penta- and hexa-nucleotide tandem repeats (*i.e.*, microsatellite loci) that met the following criteria: a minimum pattern length of 22 bp, at least 11 repeat units in case of mono-nucleotide and at least 11, 8, 6, 5, and 4 repeat units for di-, tri-, tetra-, penta- and hexa-nucleotide SSR, respectively, using the SSRFinder program [42]. Each simple sequence was counted on one strand only, and the microsatellite loci were then sorted by the monomer sequence of the repeat (e.g., AG or AAG repeats) and by the number of tandemly repeated units. Non-unique repeat motifs (reverse-complement repeat motifs (e.g., AC and GT) and translated or shifted motifs (e.g., AAT, ATA, TAA, TTA, TAT and ATT)) were grouped together, so that there were a total of 2 unique 1mer repeats, 4 unique 2mer repeats, 10 unique 3mer repeats, 33 unique 4mer, 102 unique 5mer and 350 unique 6mer repeats [43].

### 4.5. Screening of Loci suitable for PCR and Primer Design

Newly identified microsatellite loci are typically useful only if primers in the non-repeated flanking regions around the microsatellite can be designed and used successfully for PCR amplification. We therefore screened the assembled sequence with microsatellite loci for flanking regions with high quality PCR priming sites; we referred to such loci as “potentially amplifiable loci” or PAL. The primer-pair design process was automated to submit large batches of sequences to a local installation of the program Primer 3 [42]. We used fairly stringent criteria for the primer design, including the following specifications: (i) GC content >40%; (ii) melting temperatures (Tm) 60–65 °C with a maximum of 1 °C difference between paired primers; (iii) amplicon length range 80–300 bp, and (iv) primer size 24 ± 4 bp. All the remaining parameters were left at the default settings. If all criteria were met, a single primer-pair was chosen based on the highest Primer 3 assigned score and targeting the longest microsatellite element within a sequence.

### 4.6. SSR Marker Validation and Population Genetic Analysis

A subset of 100 primer pairs was synthesized and screened for amplification quality using the genomic DNA of a panel of five wild individuals. From the primers that showed scorable amplification, those that also produced specific amplification products and amplified consistently across individuals were further evaluated for marker polymorphism with additional 30 wild individual organisms sampled from the Yangtze River in China. Standard PCR was carried out in a 10 μL reaction containing 1 μL of DNA (~10 ng), 0.5 μL of forward primer and 0.5 μL of reverse primer (10 μM each), 5 μL of 2× Taq PCR MasterMix (Shanghai Xufei Company, China), and 3 μL of distilled water. The temperature cycling conditions were as follows: 95 °C for 4 min followed by 35 cycles of 94 °C for 30 s, 1 min at the annealing temperature listed in Table 2 and 72 °C for 1 min, with a final extension of 72 °C for 10 min. The separation of alleles was performed on 8% denaturing polyacrylamide gels with a 50 bp DNA marker (TaKaRa) to calculate the length of the SSR amplicons. Gels were stained with silver nitrate as previously described [44]. The allelic determination was made manually with the software package of Gel-Pro Analyzer 4.5 (http://www.mediacy.com/index.aspx?page=GelPro). The number of alleles per locus and heterozygosity were calculated using Arlequin version 3.0 [45]. Tests for linkage disequilibrium between pairs of loci and deviations from HWE (*p* < 0.05) were estimated using GENEPOP version 4.0 [46], and the adjusted *p*-values for both analyses were obtained using a sequential Bonferroni test for multiple comparisons. MICRO-CHECKER version 2.2.3 [47] was used to test the presence of null alleles.

## 5. Conclusions

The Solexa sequencing method was applied to the development of microsatellite markers for the Chinese mitten crab. More than 15,000 microsatellites were achieved using this method. To the best of our knowledge, this is the first time that such a large number of microsatellites have been isolated from this crab. At the same time, the results of this study clearly demonstrate that in addition to the 454 pyrosequencing technology, Solexa sequencing technology is suitable for the isolation of microsatellites for non-model animals in an efficient and cost-effective way.

## Figures and Tables

**Table 1:** Frequency of simple sequence repeats (SSRs) in *Eriocheir sinensis.*

Motif length	Repeat numbers												Total number	%
	4–5	6–7	8–9	10–11	12–13	14–15	16–17	18–19	20–21	22–23	24–25	>25		
Mono-										184	100	149	433	0.31
Di-				38990	15208	6239	3541	3052	2830	2441	2070	8608	82979	58.54
Tri-			30358	7936	2189	969	497	264	151	73	46	192	42675	30.11
Tetra-		9278	1093	208	61	8	2	6	1	7	3	1	10668	7.53
Penta-	2403	909	125	33	16	3	2	2	1	1		1	3496	2.47
Hexa-	1460	23	2	1									1486	.05
Total	3863	10210	31578	47168	17474	7219	4042	3324	2983	2706	2219	8951	141737	100
%	2.73	7.20	22.28	33.28	12.33	5.09	2.85	2.35	2.10	1.91	1.57	6.32		

**Table 2:** Characteristics of 20 microsatellite loci in *Eriocheir sinensis*, tested with 30 samples.

Locus	Primer sequence (5′–3′)	Repeat motif	*T*a a	Number of alleles	Allele size range (bp) b	*H*_O_	*H*_E_	*P*^c^	GeneBank Accession No.
*Eri*1	F:GATAGACCGTAAATGAGACGGCTG R:GGACGGAGAAAACTAGAGACCACA	(GGA)9	63	8	151–174 (157)	0.643	0.759	0.130	KC143114
*Eri*2	F:GGATTTACTTAAGTTGGGGCTCGT R:CGACGCAGTTTTGTCTAGAGACCT	(GAG)8	63	10	119–161 (146)	0.750	0.918	0.057	KC143100
*Eri*3^d^	F:CAGCGAAAAACAGGAAGCATTTAG R:GGAAAGGGAAAGTGAAGGATGAAT	(AC)11	63	12	140–212 (166)	0.793	0.950	0.000	KC143117
*Eri*4	F:TTCTTTGAGCGACATGCAAAAGT R:AGACAGACAGACAAAAACGCTCCT	(TG)29	62	14	132–192 (150)	0.950	0.971	0.100	KC143110
*Eri*5	F:TAGGGGGTTTTAGGTGTGGTGATA R:ATTTATGTGGAGGGAATGGGAGAT	(TGA)9	62	8	125–167 (143)	0.658	0.855	0.361	KC143115
*Eri*6^d^	F:CAACCACTACAACTATCAAAACCACC R:GACTTTACGACCACGAAATGGAG	(CA)26	62	9	91–181 (109)	0.792	0.897	0.151	KC143108
*Eri*7	F:TAACCTAAACAGCAACAGCAGCAA R:AAAGGGTTAGAAAGGAAGGAGGGT	(AGT)8	63	4	128–146 (134)	0.567	0.693	0.251	KC143118
*Eri*8^d^	F:TGTTGAGTGTGATGTTTGTGATGC R:TAATAGCGGCCAAACTTTGTTGAT	(GT)12	65	11	160–226 (196)	0.958	0.935	0.001	KC143116
*Eri*9	F:TGCATATTGTTGTTTTTACTGACGTGT R:CATCATCACCATCATCATCACAAA	(AC)18	63	12	140–188 (154)	0.884	0.945	0.641	KC143109
*Eri*10	F:TACCTTTTTCAGGGTGAGTGAAGG R:AAGGACAGGAGGGAAAATGAGAGT	(GT)23	63	7	138–208 (160)	0.865	0.917	0.317	KC143102
*Eri*11	F:ATGTTTATTTTCACAACGCGAAGC R:TGTCTTCCTTGTCTCTGTCTGTGTG	(ACAT)6	63	4	157–177 (157)	0.583	0.732	0.002	KC143103
*Eri*12	F:ACCCATCTCAAGTCCAGACTCATC R:AGAGGATGCAAGGGAAATAAGGAG	(CCT)8	63	7	152–170 (161)	0.700	0.784	0.113	KC143113
*Eri*13	F:AAGGAAGGCAGTTAGGAGGGTATG R:TTATTATTGTGGCGACGAAGGGT	(AC)11	65	8	189–259 (221)	0.668	0.950	0.147	KC143106
*Eri*14^d^	F:TGTTGTGTTGTCATGTCTTGTCTTTT R:TCAGAAACACCGCACTCGATATAA	(TCTCA)5	62	2	142–147 (142)	0.420	0.510	0.000	KC143119
*Eri*15	F:TCACCCCTTACTGAGCATAACACA R:CCTTATCCTGCGACTCGTAATGTT	(CA)12	63	5	93–109 (105)	0.736	0.773	0.250	KC143105
*Eri*16^d^	F:TCCTCCCTATGCTCTTTGTAGGTG R:AAGGCCCAGGAGTATGGTGAAC	(AC)11	63	8	150–192 (156)	0.699	0.786	0.115	KC143111
*Eri*17	F:CAGCATGTCCAGTCTCTTCTGTGT R:GCTGAGAGAATATGTATGATGACATGG	(GT)15	65	6	141–159 (145)	0.772	0.864	0.054	KC143107
*Eri*18	F: TGGCATTGATTGATGTGAGTAGTG R:CTAACCTTCTCGACACCTTTGCAT	(GGT)10	62	5	85–106 (94)	0.326	0.551	0.269	KC143104
*Eri*19	F:CAGACCCTCCCGATGATACACTAC R:CTATCCACTCAGCTACCGCCTCT	(ATCT)8	63	4	133–155 (143)	0.563	0.668	0.531	KC143112
*Eri2*0	F: GAGATGGAGGTAGATGATCGAGGA R:CAAGGCACTCAATCTCAACCTTTT	(GAAG)7	63	4	120–144 (132)	0.447	0.532	0.516	KC143101

a annealing temperature;

b allele size (size of sequenced allele);

c *p*-values of test for deviation from Hardy-Weinberg equilibrium;

d microsatellite loci revealed the presence of null alleles with MICRO-CHEKER 2.2.3.

## References

[1] Weber J.L. (1990). Informativeness of human (dC-dA)n.(dG-dT)n polymorphisms. Genomics.

[2] Field D., Wills C. (1996). Long, polymorphic microsatellites in simple organisms. Proc. R. Soc. Lond. Ser. B.

[3] Goldstein D.B., Schlotterer C (1999). Microsatellites: Evolution and Applications.

[4] Gupta P., Varshney R. (2000). The development and use of microsatellite markers for genetic analysis and plant breeding with emphasis on bread wheat. Euphytica.

[5] Gu Z.X., Gou T.J., Xi X.B. (2006). Applications of microsatellite markers in studies of genetics and breeding of fish. Chin. J. Agric. Biotechnol.

[6] Chistiakov D.A., Hellemans B., Volckaert F.A.M. (2006). Microsatellites and their genomic distribution, evolution, function and applications: A review with special reference to fish genetics. Aquaculture.

[7] Zane L., Bargelloni L., Patarnello T. (2002). Strategies for microsatellite isolation: A review. Mol. Ecol.

[8] Yu J.-N., Won C., Jun J., Lim Y., Kwak M. (2011). Fast and Cost-Effective Mining of Microsatellite Markers Using NGS Technology: An Example of a Korean Water Deer *Hydropotes inermis argyropus*. PLoS One.

[9] Castoe T.A., Poole A.W., Gu W., de Koing A.P.J., Daza J.M., Smith E.N., Pollock D.D. (2010). Rapid identification of thousands of copperhead snake (*Agkistrodon contortrix*) microsatellite loci from modest amounts of 454 shotgun genome sequence. Mol. Ecol. Resour.

[10] Jennings T.N., Knaus B.J., Mullins T.D., Haig S.M., Cronn R.C. (2011). Multiplexed microsatellite recovery using massively parallel sequencing. Mol. Ecol. Resour.

[11] Blanca J., Canizares J., Roig C., Ziarsolo P., Nuez F., Pico B. (2011). Transcriptome characterization and high throughput SSRs and SNPs discovery in *Cucurbita pepo* (Cucurbitaceae). BMC Genomics.

[12] Setsuko S., Uchiyama K., Sugai K., Yoshimaru H. (2012). Rapid development of microsatellite markers for *Pandanus boninensis* (Pandanaceae) by pyrosequencing technology. Am. J. Bot.

[13] Saarinen E.V., Austin J.D. (2010). When Technology Meets Conservation: Increased Microsatellite Marker Production Using 454 Genome Sequencing on the Endangered Okaloosa Darter (*Etheostoma okaloosae*). J. Hered.

[14] Kang J.H., Park J.Y., Jo H.S. (2012). Rapid Development of Microsatellite Markers with 454 Pyrosequencing in a Vulnerable Fish, the Mottled Skate, Raja pulchra. Int. J. Mol. Sci.

[15] Wang J., Yu X., Zhao K., Zhang Y., Tong J., Peng Z. (2012). Microsatellite development for an endangered bream Megalobrama pellegrini (Teleostei, Cyprinidae) using 454 sequencing. Int. J. Mol. Sci.

[16] Fushun G.X.Z. (2001). Resources and Culturing Situation of Chinese Mitten Crab ( *Eriocheir sinensis*) and Species Character Conservation. J. Lake Sci.

[17] Food and Agriculture Organization of the United Nations (2010). FAO Yearbooks of Fishery Statistics Summary Tables, Aquaculture Production 2009.

[18] Ozaki A., Sakamoto T., Khoo S., Nakamura K., Coimbra M.R.M., Akutsu T., Okamoto N. (2001). Quantitative trait loci (QTLs) associated with resistance/susceptibility to infectious pancreatic necrosis virus (IPNV) in rainbow trout (*Oncorhynchus mykiss*). Mol. Genet. Genomics.

[19] Dekkers J.C.M., Hospital F. (2002). The use of molecular genetics in the improvement of agricultural populations. Nat. Rev. Genet.

[20] Du N.S., Lai W., Xue L.Z. (1986). The chromosomes of the Chinese mitten-handed crab, *Eriocheir sinensis* (Crustacea, Decapoda). Zool. Res.

[21] Hanfling B., Weetman D. (2003). Characterization of microsatellite loci for the Chinese mitten crab, *Eriocheir sinensis*. Mol. Ecol. Notes.

[22] Zhu Z.Y., Shi Y.H., Le G.W. (2006). Isolation and characterization of polymorphic microsatellites from Chinese mitten crab, *Eriocheir sinensis*. Mol. Ecol. Notes.

[23] Mao R.X., Zhao Y.Y., Liu F.J., Jia Z.Y., Hou N., Chang Y.M., Lu C.Y., Liang L.Q., Sun X.W. (2009). Development and characterization of new microsatellite loci from Chinese mitten crab (*Eriocheir sinensis*). Conserv. Genet.

[24] Chang Y.M., Liang L.Q., Li S.W., Ma H.T., He J.G., Sun X.W. (2006). A set of new microsatellite loci isolated from Chinese mitten crab, *Eriocheir sinensis*. Mol. Ecol. Notes.

[25] Li R., Yu C., Li Y., Lam T.-W., Yiu S.-M., Kristiansen K., Wang J. (2009). SOAP2: An improved ultrafast tool for short read alignment. Bioinformatics (Oxford).

[26] Cheng Q., Yuan C., Wang J., Xu J., Lee T.-H., Wang C. (2010). Development of 20 microsatellite loci in the Japanese mitten crab *Eriocheir japonica* and cross-amplification in the Chinese mitten crab *Eriocheir sinensis*. Conserv. Genet. Resour.

[27] Bachtrog D., Agis M., Imhof M., Schloetterer C. (2000). Microsatellite variability differs between dinucleotide repeat motifs: Evidence from *Drosophila melanogaster*. Mol. Biol. Evol.

[28] Lecher P., Defaye D., Noel P. (1995). Chromosomes and nuclear DNA of Crustacea. Invertebr. Reprod. Dev.

[29] Kong J., Gao H.A. (2005). Analysis of tandem repeats in the genome of Chinese shrimp *Fenneropenaeus chinensis*. Chin. Sci. Bull.

[30] Schug M.D., Wetterstrand K.A., Gaudette M.S., Lim R.H., Hutter C.M., Aquadro C.F. (1998). The distribution and frequency of microsatellite loci in *Drosophila melanogaster*. Mol. Ecol.

[31] Edwards Y.J.K., Elgar G., Clark M.S., Bishop M.J. (1998). The identification and characterization of microsatellites in the compact genome of the Japanese pufferfish, *Fugu rubripes*: Perspectives in functional and comparative genomic analyses. J. Mol. Biol.

[32] Lander E.S., Linton L.M., Birren B., Nusbaum C., Zody M.C., Baldwin J., Devon K., Dewar K., Doyle M., FitzHugh W. (2001). Initial sequencing and analysis of the human genome. Nature (Lond.).

[33] Wang Z., Weber J.L., Zhong G., Tanksley S.D. (1994). Survey of plant short tandem DNA repeats. Theor. Appl. Genet.

[34] Van Belkum A., Scherer S., van Alphen L., Verbrugh H. (1998). Short-sequence DNA repeats in prokaryotic genomes. Microbiol. Mol. Biol. Rev.

[35] Ross C.L., Dyer K.A., Erez T., Miller S.J., Jaenike J., Markow T.A. (2003). Rapid divergence of microsatellite abundance among species of Drosophila. Mol. Biol. Evol.

[36] Lagercrantz U., Ellegren H., Andersson L. (1993). The abundance of various polymorphic microsatellite motifs differs between plants and vertebrates. Nucleic Acids Res.

[37] Toth G., Gaspari Z., Jurka J. (2000). Microsatellites in different eukaryotic genomes: Survey and analysis. Genome Res.

[38] Chang Y., Liang L., Ma H., He J., Sun X. (2008). Microsatellite analysis of genetic diversity and population structure of Chinese mitten crab (*Eriocheir sinensis*). J. Genet. Genomics.

[39] Herborg L.-M., Weetman D., Van Oosterhout C., Hanfling B. (2007). Genetic population structure and contemporary dispersal patterns of a recent European invader, the Chinese mitten crab, *Eriocheir sinensis*. Mol. Ecol.

[40] Sambrook J., Russell D.W. (2001). Molecular Cloning: A Laboratory Manual.

[41] Pevzner P.A., Tang H.X., Waterman M.S. (2001). An Eulerian path approach to DNA fragment assembly. Proc. Natl. Acad. Sci. USA.

[42] Rozen S., Skaletsky H. (2000). Primer3 on the WWW for general users and for biologist programmers. Methods Mol. Biol.

[43] Jurka J., Pethiyagoda C. (1995). Simple receptive DNA sequences from primates: Compilation and analysis. J. Mol. Evol.

[44] Bassam B.J., Caetanoanolles G., Gresshoff P.M. (1991). Fast and sensitive silver staining of DNA in polyacrylamide gels. Anal. Biochem.

[45] Excoffier L., Lischer H.E.L. (2010). Arlequin suite ver 3.5: A new series of programs to perform population genetics analyses under Linux and Windows. Mol. Ecol. Resour.

[46] Rousset F. (2008). GENEPOP’007: A complete re-implementation of the GENEPOP software for Windows and Linux. Mol. Ecol. Resour.

[47] Van Oosterhout C., Hutchinson W.F., Wills D.P.M., Shipley P. (2004). MICRO-CHECKER: Software for identifying and correcting genotyping errors in microsatellite data. Mol. Ecol. Notes.

